# Development of Carbon Nanotube Modified Cement Paste with Microencapsulated Phase-Change Material for Structural–Functional Integrated Application

**DOI:** 10.3390/ijms16048027

**Published:** 2015-04-10

**Authors:** Hongzhi Cui, Shuqing Yang, Shazim Ali Memon

**Affiliations:** 1 Guangdong Provincial Key Laboratory of Durability for Marine Civil Engineering, College of Civil Engineering, Shenzhen University, Shenzhen 518060, China; 2 Department of Civil and Environmental Engineering, School of Engineering, The Hong Kong University of Science and Technology, Hong Kong 999077, China; E-Mail: syangaq@connect.ust.hk; 3 Department of Civil Engineering, COMSATS Institute of Information Technology, Abbottabad Campus, Abbottabad 22010, Pakistan

**Keywords:** carbon nanotube, cement paste, microencapsulated phase-change material, structural–functional integrated materials, mechanical properties, thermal energy storage

## Abstract

Microencapsulated phase-change materials (MPCM) can be used to develop a structural–functional integrated cement paste having high heat storage efficiency and suitable mechanical strength. However, the incorporation of MPCM has been found to degrade the mechanical properties of cement based composites. Therefore, in this research, the effect of carbon nanotubes (CNTs) on the properties of MPCM cement paste was evaluated. Test results showed that the incorporation of CNTs in MPCM cement paste accelerated the cement hydration reaction. SEM micrograph showed that CNTs were tightly attached to the cement hydration products. At the age of 28 days, the percentage increase in flexural and compressive strength with different dosage of CNTs was found to be up to 41% and 5% respectively. The optimum dosage of CNTs incorporated in MPCM cement paste was found to be 0.5 wt %. From the thermal performance test, it was found that the cement paste panels incorporated with different percentages of MPCM reduced the temperature measured at the center of the room by up to 4.6 °C. Inverse relationship was found between maximum temperature measured at the center of the room and the dosage of MPCM.

## 1. Introduction

Cement based composite is an important and versatile building material in every area of construction worldwide. As structural materials, cementitious materials are quasi-brittle, susceptible to cracking [1,2] and have no functional properties. A structural–functional integrated cement-based material means that the material serves both as a structural material for building applications and as a functional material for thermal energy storage applications [3]. The technology of utilizing phase-change materials (PCMs) to store and retrieve latent heat has been considered a simple and effective technique for application to building envelopes to enhance the energy efficiency of buildings [4,5]. However, PCM must be encapsulated before it can be utilized in cement based composites.

Microencapsulation is a technology in which PCM particles are enclosed in a thin and high-molecular-weight polymeric film to prevent the leakage of PCM during the phase-change process [6,7]. In addition, microencapsulated PCM (MPCM) provides a high heat transfer rate through its larger surface area per unit volume and is capable of resisting volume change during phase transition. For many applications, paraffins have been microencapsulated and used by many researchers [8,9]. However, it is known that the addition of MPCM degrades the mechanical properties of structural–functional integrated composite materials. Cui *et al.* [10] experimentally investigated the effect of the incorporation of different percentages of microencapsulated PCM (5%, 10%, 15% and 20% by cement mass) on the material properties of cement paste. The incorporation of microencapsulated PCM was found to effectively improve the thermal properties of the cement based composite. However, a reduction in compressive and flexural strength of up to 67% was obtained with the inclusion of 20% PCM.

Cement based composites can be nano-engineered by incorporation of these CNTs. In spite of the high cost of CNTs, it is still being used not only to improve the mechanical properties and durability of cementitious materials but it also endues them with such functionalities as electrical, thermal and electromagnetic properties [1,2]. CNTs have been used for fabricating piezoresistive strain sensing cement-based composites [11,12,13,14]. According to the number of rolled layers of graphene, CNTs are categorized as single-walled CNTs (SWCNTs) and multi-walled CNTs (MWCNTs) [15,16]. These CNTs are generally a few nanometers in diameter and several microns in length, therefore, for cement based composites, CNTs can provide more obvious improvement in flexural strength than particle additives. Hence, in this research, we investigated the effect MWCNTs on the properties of cement paste MPCM to obtain a structural–functional integrated cement-based material. The effect of CNT on heat of hydration of cement paste and mechanical properties is evaluated. Moreover, the thermal performance of cement based panels with CNTs and containing different percentage of MPCM is determined.

## 2. Results and Discussion

### 2.1. Effect of CNTs on Cement Hydration

**Figure 1:**
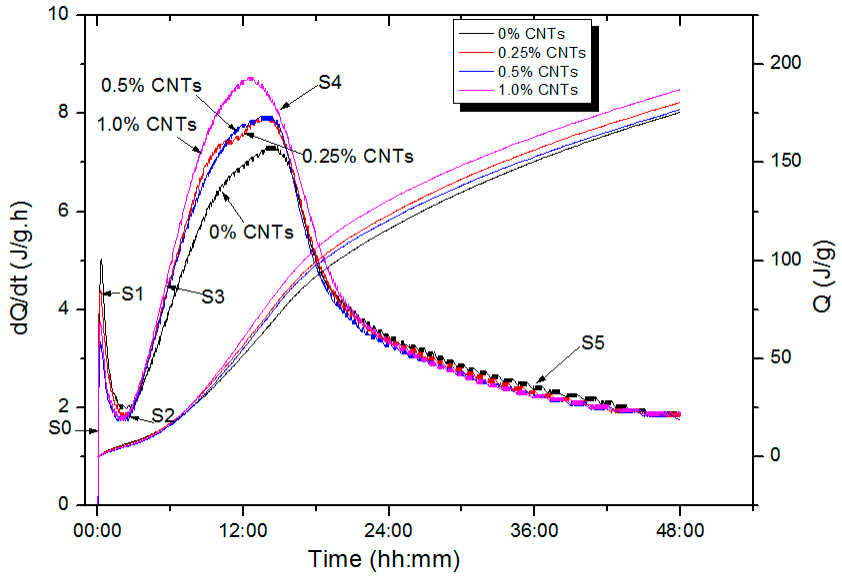
Heat flow calorimetry of 10%-microencapsulated phase change materials cement paste (MPCM-CP) with different dosages of carbon nanotubes (CNT).

The heat of hydration of MPCM cement paste with different percentages of CNTs was observed so as to evaluate the effect of CNTs on the heat of hydration of cement paste. The hydration heat releasing rate dQ/dt of cement paste with 10%-MPCM-CP and containing different dosage of CNTs in the first 48 h of hydration are shown in Figure 1. There are six stages depicted in the curves of dQ/dt [17]: (S0) rapid dissolution, (S1) first deceleration, (S2) induction or dormancy, (S3) acceleration, (S4) second deceleration, and (S5) slow continued reaction.

During the first three hours (pre-acceleration stage), the hydration rates of MPCM-CPs with dosage of 0.25%, 0.5%, 1% CNTs are close to control MPCM-CP. At the stage of acceleration, the heat of hydration of all the samples was very similar. Moreover, there is no evidence that CNTs have great influence on cement hydration at the stages of the first deceleration and induction.

Starting from about 3 h, all samples began to enter the acceleration period. At this stage, the heat mainly come from C_3_S (3CaO·SiO_2_) hydration and the formations of C–S–H and ettringite [17]. The cement hydration rate of MPCM-CP with 1% CNTs was greater than the hydration rate of the other groups and reached the peak at about 12 h. It can also be seen that the hydrate rate of MPCM-CP without CNTs was the lowest and reached the peak at about 13 h. Therefore, carbon nanotubes obviously accelerate the cement hydration reaction. Makar and Chan [18] studied the effect of 1% SWCNTs on cement hydration and reported that CNTs accelerated the cement hydration rate. The authors also carried out SEM investigations and found SWCNTs didn’t affect the morphology of C_3_A (3CaO·Al_2_O_3_). In [19], Marker and Margeson utilized Vickers hard to evaluate the effect of carbon nanotubes on early hydration of cement. They found that carbon nanotubes have accelerating effect on cement hydration. It was shown that different content of CNTs resulted in different final setting time. Moreover, the final setting time of cement paste with CNTs was always less than that of the control cement paste without CNT. Therefore, the incorporation of CNTs can shorten setting time of cement paste. Researches [20,21] have found that the addition of CNTs was helpful in dispersing the cement particles, which in turn, leads to accelerate the early hydration process of the cement. Makar and Chan [18] think that polarization and adsorption between cement particles and CNTs leads to accelerate the early hydration of cement. Because of the nano size effect, CNTs added in cements may serve as nucleation sites for the hydration products. The earlier these nuclei are formed, the earlier they can grow with the hydration phases and thereby accelerate the cement hydration. Moreover, CNTs having very large surface areas are highly reactive and may react with components from the pore solution. We think that the mechanism for effect CNTs on cement hydration is similar to the model to describe the influence of nanoparticles on cement hydration as mentioned in [22].

**Figure 2:**
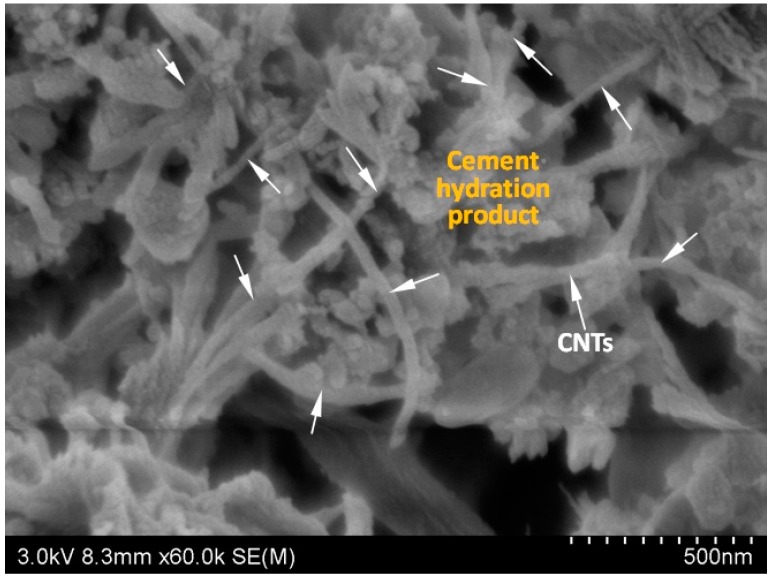
Microstructure of MPCM-CP with 0.25% CNTs (cement hydrated 12 h).

In order to explore the microstructure of CNTs cement based composite, the micrograph captured by SEM is shown in Figure 2. It can be seen that the cement hydration products are tightly attached to the surface of carbon nanotubes. Therefore, CNTs can disperse cement particles and provide nucleation sites for cement hydration products. This provides evidence that adding CNTs promote the early cement hydration. Moreover, due to the large aspect ratio of CNTs, it bridges across pores and cement hydration products (Figure 2). Because of the nano size effect of CNTs, it should also serve as filler resulting in a dense microstructure and enhanced mechanical properties when compared to control cement paste without CNTs.

### 2.2. Effect of CNTs on Mechanical Properties of Microencapsulated Phase Change Material Cement Paste (MPCM-CP)

CNTs addition can enhance the mechanical properties of cement based materials mainly due to fiber bridging effect, packing effect resulting from small CNT, the cement hydration acceleration, *etc.* [2,23,24,25]. The flexural and compressive strength results of microencapsulated PCM with different percentages of CNT are shown in Table 1.

**Table 1:** Mechanical properties of CNTs modified MPCM-CP.

**Type of Cement Paste**	**3 Days Flexural Strength/MPa**	**Increase (%)**	**3 Days Compressive Strength/MPa**	**Increase (%)**
MPCM-CP-0% (control)	1.92	0.0	10.4	0.0
MPCM-CP-0.25%	2.31	20.3%	10.8	3.8%
MPCM-CP-0.5%	2.53	31.8%	11.2	7.7%
MPCM-CP-1.0%	2.43	26.6%	10.5	1.0%
**Type of Cement Paste**	**28 Days Flexural Strength/MPa**	**Increase (%)**	**28 Days Compressive Strength/MPa**	**Increase (%)**
MPCM-CP-0% (control)	4.22	0.0	27.1	0.0
MPCM-CP-0.25%	5.25	24.4%	28.4	4.8%
MPCM-CP-0.5%	5.94	40.8%	27.9	3.0%
MPCM-CP-1.0%	5.39	27.7%	27.6	1.8%

In comparison to control MPCM-CP with 0% CNT, the flexural strength increased with the addition of CNT. At the age of 3 and 7 days, the percentage increase in all CNT samples was found to be higher than 20%. Moreover, in comparison to control MPCM-CP, the MPCM-CP with 0.5% CNTs showed the highest percentage increase in flexural strength at both the ages of testing. This sample showed the percentage increase of 31.8% and 40% at the age of 3 and 28 days. The results of flexural strength are consistent with the results reported in literatures [21,26,27] where they showed that the strength of cement paste incorporated with CNTs was found to be higher than pure cement. However, the increase in CNT dosage would not always lead to increase in the strength of the resulting cement paste. The possible reasons are (1) after reaching the optimal dosage; the strength decreases possibly due to agglomeration of CNT which induces local stress concentrations that affect strength [28]. The experiment results reported in literature [23] have also observed this phenomenon; (2) since the aspect ratio of carbon nanotubes is very large therefore it is not easy to disperse them in the cement based materials. Therefore, the dispersion of CNTs in hardened cement paste should be observed carefully when the amount of CNT in hardened cement paste is increased. For this research, the incorporation of 0.5% CNT was found to be optimum.

As far as the compressive strength results are concerned, the maximum percentage increase with different percentage of CNTs was found to be 7.7% at the age of 3 days and 4.8% at the age of 28 days. In comparison to flexural strength, the increase in the percentage of compressive strength was found to be lower. The experimental results are consistent with the results reported in literature [25] where they found that the percentage increase in flexural strength was higher than the percentage increase in compressive strength. Moreover, due to larger aspect ratio of CNTs, larger bond area in cement paste can be obtained, which lead to better bond between CNT and the cement paste. Therefore, good load-transfer mechanism especially in tension is ensured [29]. It is worth mentioning here that the maximum percentage increase in compressive strength of cement paste with 0.5–1 wt % was about 10% [30,31,32].

### 2.3. Effect of CNTs on Thermal Performance of MPCM-CP

**Figure 3:**
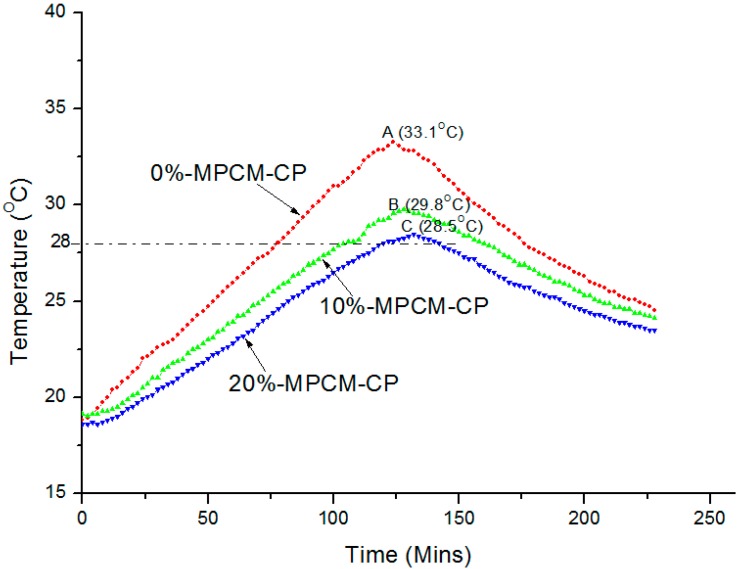
Thermal performance comparison between control and different MPCM-CPs with 0.5% CNT.

The thermal performance of MPCM-CP is reflected by the thermal performance of MPCM-CP working as a wall of the small test room model. The thermal performance of the control and MPCM-CP room model was evaluated by monitoring the temperature variation at the center of the test room during the test period of approximately 4 h. The temperature variation curves of the room model with the top panel prepared with control and different percentages of MPCM (10% and 20%) containing 0.5% CNT are shown in Figure 3. The maximum temperature values at the center of the room were found to be 33.1 °C (A), 29.8 °C (B) and 28.5 °C (C) for the control, 10%-MPCM-CP and 20%-MPCM-CP. This shows that the maximum temperature measured at the center of the room model has an inverse relationship with the dosage of MPCM. The difference in the maximum indoor center temperature between control and 10%-MPCM-CP is 3.3 °C while this difference is 4.6 °C in between the control and 20%-MPCM-CP. Thus, in can be deduced that the part of the heating load has been taken by microencapsulated PCM. Li *et al.* [33] reported maximum indoor air temperature differences of 2.2 °C when 20 wt % cement was replaced by the paraffin/expanded graphite composite PCM. Moreover, according to the authors, the heat storage coefficient of the 30-mm heat storage cement mortar board with 20 wt % composite PCM was found to be 1.74 times more than that of the ordinary cement mortar board.

Figure 3 shows that the peak temperatures for the room model with different percentages of MPCM are right-shifted and have a flatter profile. The delay in the peak temperature with 20%-MPCM-CP was about 12 min when compared with the control room model. It is known that the consumption of electricity varies during the day and night according to the demand by building, industrial and commercial sectors. This variation can lead to a differential pricing system for peak and off peak periods.

Thus, MPCM-CP can be beneficial in such situation by shifting the load away from the peak demand times [34,35,36]. Hence, energy can be purchased at a lower cost during off-peak periods. It can also be observed (Figure 3) that the indoor centre temperature of control room model increases almost steadily in the heating process. In contrast, the temperature curves for the MPCM-CP room models show a little hump when the temperature reaches approximate 28 °C. This show that phase change has occurred in the PCM.

Based on above observations and discussion, it can be concluded that cement paste panels with different percentages of MPCM has a function of reducing the energy consumption by decreasing the temperature and shifting the loads away from the peak periods and hence have promising applications in buildings.

## 3. Experimental Section

### 3.1. Materials

**Figure 4:**
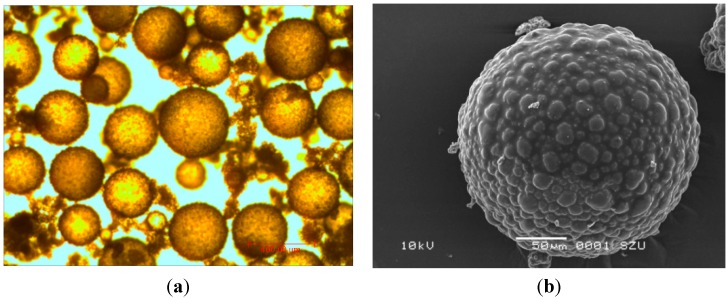
The morphology of manufactured MPCM (**a**) optical microcopy; (**b**) SEM.

An industrial grade hydroxyl multi-wall carbon nanotubes purchased from The Physics Institute of Chinese Academy of Sciences was used. The properties of MWCNT are enlisted in Table 2. The microencapsulated PCM used in this research was prepared with the same method as reported in literature [10]. The microcapsule core contained a type of paraffin having phase change temperature of 28 °C and latent heat storage capacity of 206 J/g. The morphology of manufactured MPCM examined by optical microcopy and scanning electron microscopy (SEM) is shown in Figure 4. The cement used for this research was P.II 42.5R Portland cement complying with the requirements of GB 175-2007 (China National Standard: Common Cement Paste) while the polycarboxylate superplasticizer was used to adjust workability of the cement pastes.

**Table 2:** Properties of MWCNT.

Items	Data
Outer Diameter	20~50 nm
–OH Content	0.51%
Length	10~30 μm
Purity	>90 wt %
Ash	<8 wt %
Specific Surface Area (SSA)	40 m^2^/g
Electrical Conductivity (EC)	>10^2^ s/cm

### 3.2. MWCNT Dispersion Method

Since CNTs have a strong tendency to cluster due to van der Waals forces therefore it is difficult to disperse them uniformly. Many researchers [2] have used sonication method to disperse CNTs. Azhari and Banthia [11] used both sonication and dry mix methods to disperse CNTs in cement paste. According to the authors, the two dispersion methods produced similar resistivity values. Therefore, the dispersion degrees of two methods are similar.

In this research, a simple and effective two-step dispersion strategy was employed. At first, CNTs were added to cement and mixed in dry state for 5 min using cement paste mixing machine at high speed. The dry mixing at high speed helps the CNTs to disperse uniformly in the cement powder. Thereafter, water and superplasticizer were added to the dry mixture and the mixing was continued for another 10 min. Since the CNTs used in this research were surface treated with hydroxyl functional group, therefore, the superplasticizer (a surfactant) can help CNTs to disperse more effectively in fresh cement paste. Moreover, the longer mixing time during second stage ensures that the CNTs can be dispersed uniformly in cement paste.

### 3.3. Test Methods along with the Details of Mix Design

#### 3.3.1. Heat of Hydration of Microencapsulated PCM Cement Paste with CNTs

In order to study the effect of CNTs on the heat of hydration heat of cement paste, the cement paste with 10% MPCM and containing different percentages of CNTs (0.25%, 0.5% and 1.0 wt %) were prepared with constant water cement ratio of 0.35. The details of the mix design are shown in Table 3. The heat of hydration of cement paste in the first 48 h of hydration was measured using a ToniCal Trio 7338 instrument (Toni Technik, Zwick/Roell Group, Berlin, Germany).

**Table 3:** Details of mix proportion.

Heat of Hydration Test					
Type of Cement Paste	Cement	Water	CNTs	MPCM	Superplasticizer
* MPCM-CP-0% (control)	1	0.35	0%	10%	/
MPCM-CP-0.25%	1	0.35	0.25%	10%	/
MPCM-CP-0.5%	1	0.35	0.5%	10%	/
MPCM-CP-1.0%	1	0.35	1.0%	10%	/
**Compressive and** **Flexural Strength Test**					
MPCM-CP-0% (control)	1	0.35	0%	15%	0.40%
MPCM-CP-0.25%	1	0.35	0.25%	15%	0.50%
MPCM-CP-0.5%	1	0.35	0.5%	15%	0.73%
MPCM-CP-1.0%	1	0.35	1.0%	15%	0.80%
**Thermal Performance Test**					
^ 0%-MPCM-CP (control)	1	0.35	0.5%	0%	0.39%
10%-MPCM-CP	1	0.35	0.5%	10%	0.58%
15%-MPCM-CP	1	0.35	0.5%	15%	0.73%
20%-MPCM-CP	1	0.35	0.5%	20%	0.81%

MPCM-CP = Microencapsulated Phase change material cement paste; * MPCM-CP-X = MPCM cement paste with different % of carbon nanotubes; ^ Y-MPCM-CP = MPCM cement paste with different % of MPCM at constant % of carbon nanotubes content.

#### 3.3.2. Mechanical Properties of MPCM-CP with Different Percentage of CNTs

The mechanical properties (flexural and compressive strength) of cement paste with 15% MPCM and containing different percentages of CNTs (0.25%, 0.5% and 1.0 wt %) were evaluated at the age of 3 and 28 days. The compressive strength test was carried out in accordance with the standard of ISO 679:1989 and GB/T 17671-1999 (Method of testing cements-Determination of strength). For flexural strength test, the specimens with size of 40 mm × 40 mm × 160 mm were cured under standard curing condition in a curing room (temperature 20 ± 1 °C and relative humidity of 99%) and tested at the desired age. The compressive and flexural strength represents the average of three specimens. The loading rate for flexural and compressive strength were 50 ± 10 and 2400 ± 200 N/s, respectively. The details of the mix design are shown in Table 3. The superplasticizer amount was adjusted in range of 0%–1% by weight of cement so as to make sure that the cement paste specimens have similar workability.

#### 3.3.3. Scanning Electron Microscopy

The micro morphology of MPCM-CP with CNTs was examined using a JSM-5910 LV microscope. The prepared cement paste samples were cut into thin slices using a precision diamond saw. They were then further treated for preparations normally performed for SEM including vacuum drying and gold coating.

#### 3.3.4. Thermal Performance of MPCM Cement Paste Panel Containing CNTs

**Figure 5:**
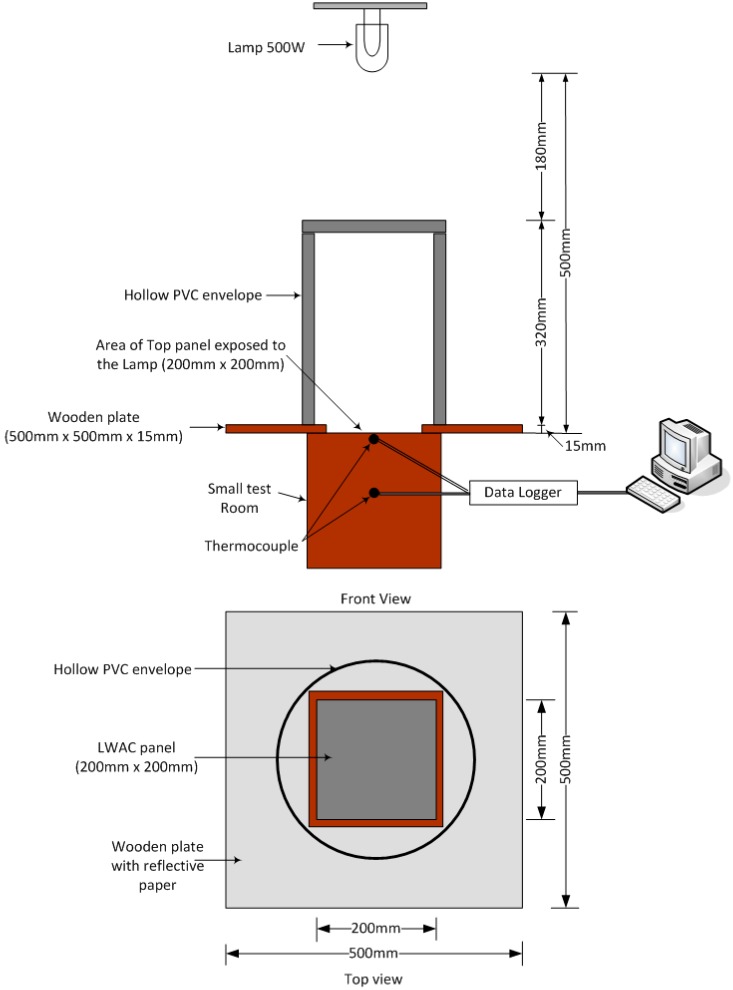
Schematic of thermal performance test–Indoor test.

The thermal performance of cement paste panels containing different percentage MPCM at constant CNT content (0.5 wt %) and having dimensions of 200 mm × 200 mm × 40 mm was evaluated at the age of 28 days using a self-designed heating system (Figure 5). The setup consisted of a small test room, a 500 W lamp (used as a heating source) placed over the top panel at a distance of 500 mm, a hollow PVC envelope with reflective paper coated inside to create a uniform and steady temperature field [37], a wooden plate (500 mm × 500 mm × 15 mm) with an opening of 200 mm × 200 mm × 15 mm placed between the PVC envelope and the test room, thermocouples (Type K resolution ±0.3 °C) placed in the centre of the test room and a computer recording system connected to a data-logger. The purpose of using the wooden plate with the top surface coated with reflective paper was to avoid heating the side panels.

The small test room consisted of six panels; out of which five panels were made of wood while the top panel (with and without MPCM) was used to evaluate the thermal performance. In this test, the top panel was heated for 2 h and was then allowed to cool naturally for approximately 2 h. The details of the mix design used for thermal performance test are given in Table 3.

## 4. Conclusions

In this research, we developed CNT modified cement paste containing microencapsulated PCM for structural–functional integrated applications. Based on the test results, the following conclusions can be drawn:

(1) The incorporation of carbon nanotubes in MPCM cement paste was obviously found to accelerate the cement hydration reaction.
(2) CNTs enhanced the flexural and compressive strength of MPCM incorporated cement paste. The percentage increase in flexural and compressive strength at the age of 28 days and with different dosage of CNTs was found to be up to approximately 41% and 5% respectively. The optimum dosage of CNTs incorporated in MPCM cement paste was found to be 0.5 wt %.
(3) From thermal performance test, an inverse relationship was found between the maximum temperature measured at the center of the room and the dosage of MPCM. The temperature decreased with the increase in the percentage of MPCM. In comparison to control room model, the reduction in maximum temperature measured at the center of the MCPM room model was as high as 4.6 °C. Moreover, the function of delaying the peak temperature of MPCM-CP was certified. Thus, the developed carbon nanotubes based composite MPCM is a potential candidate for structural–functional integrated application.
